# Quantitative proteomic analysis to reveal expression differences for butanol production from glycerol and glucose by *Clostridium* sp. strain CT7

**DOI:** 10.1186/s12934-021-01508-3

**Published:** 2021-01-09

**Authors:** Yujia Jiang, Ruofan Wu, Jiasheng Lu, Weiliang Dong, Jie Zhou, Wenming Zhang, Fengxue Xin, Min Jiang

**Affiliations:** 1 State Key Laboratory of Materials-Oriented Chemical Engineering, College of Biotechnology and Pharmaceutical Engineering, Nanjing Tech University, Puzhu South Road 30#, 211800 Nanjing, P. R. China; 2 Jiangsu National Synergetic Innovation Center for Advanced Materials (SICAM), Nanjing Tech University, 211800 Nanjing, P.R. China

**Keywords:** iTRAQ, Proteomic analysis, Glycerol, Butanol, *Clostridium*

## Abstract

*Clostridium* sp. strain CT7 is a new emerging microbial cell factory with high butanol production ratio owing to its non-traditional butanol fermentation mode with uncoupled acetone and 1,3-propanediol formation. Significant changes of metabolic products profile were shown in glycerol- and glucose-fed strain CT7, especially higher butanol and lower volatile fatty acids (VFAs) production occurred from glycerol-fed one. However, the mechanism of this interesting phenomenon was still unclear. To better elaborate the bacterial response towards glycerol and glucose, the quantitative proteomic analysis through iTRAQ strategy was performed to reveal the regulated proteomic expression levels under different substrates. Proteomics data showed that proteomic expression levels related with carbon metabolism and solvent generation under glycerol media were highly increased. In addition, the up-regulation of hydrogenases, ferredoxins and electron-transferring proteins may attribute to the internal redox balance, while the earlier triggered sporulation response in glycerol-fed media may be associated with the higher butanol production. This study will pave the way for metabolic engineering of other industrial microorganisms to obtain efficient butanol production from glycerol.

## Introduction

Due to the fluctuating prices and environmental issues caused by fossil fuels, biofuels have attracted great attention as promising alternatives [1, 2]. In particular, biobutanol is considered as a more superior biofuel to bioethanol, which possesses higher energy density, lower vapor pressure, better intersolubility and less corrosiveness [3–5]. Acetone-butanol-ethanol (ABE) fermentation is a traditional bioprocess to synthesize butanol by using solventogenic *Clostridia*, which can be traced back to 1910 s [6, 7]. Currently, the most used solventogenic clostridia are *Clostridium acetobutylicum* and *C. beijerinckii* [8]. Although these bacteria showed wide carbon utilization spectra including both pentose and hexose, however, the carbon sources are mainly limited in molasses and starchy materials [9]. The high cost of these substrates hindered the industrial butanol production, which accounts for about 50% of the total cost [10].

Glycerol is released as a by-product in the biodiesel industry with production of approximate ten percent of biodiesel in weight [11]. In particular, crude glycerol is an abundant and versatile carbon source for biochemicals production owing to its massive dropped price [12, 13]. Fascinatingly, glycerol offers more reducing power than glucose through the initial step of glycerol metabolism, which is specifically significant for some reduced products production including butanol [14]. The theoretical yield for butanol production from glycerol is 17% higher than that from glucose on a carbon-mole basis [15]. These unique features make glycerol as a promising substrate for butanol production. However, most solventogenic clostridia including *C. acetobutylicum* and *C. beijerinckii* could not efficiently utilize glycerol. Different from these traditional solventogenic strains, *C. pasteurianum*, another kind of solventogenic producers prefers glycerol as the substrate for butanol production [16]. While the butanol titer and yield were significantly decreased on account of acetone and 1,3-propanediol (PDO) generation, the major by-products when glycerol was used as the substrate [17]. Comprehensive studies in terms of elimination of by-products formation from glycerol through genetic modification or mutagenesis by using *C. pasteurianum* have been investigated. However, majority of attempts still did not end up with recombinants or mutants exhibiting similar butanol producing characteristics as the well-known *C. acetobutylicum* and *C. beijerinckii* from glucose.

In our previous studies, a unique butanologenic *Clostridium* sp. strain CT7 was successfully isolated with efficient butanol production capability from glycerol. Moreover, due to the indigenous elimination of acetone and 1,3-PDO, strain CT7 could synthesize butanol with high ratio of 94%, thereby providing a quantum leap towards establishing sustainable strategies for bioconversion of waste glycerol into butanol [18]. To further elaborate the underlying mechanism, an isobaric tag for relative and absolute quantitation (iTRAQ™) based proteomics strategy of strain CT7 was carried out from different growth phases fed with glycerol and glucose, respectively. The comparative proteomic analysis of key proteins related with substrates utilization, solvents and volatile fatty acids (VFAs) generation were analyzed. In addition, changes of expression levels in major proteins relevant to electron-transferring, energy metabolism and stress response were also discussed. These results provided evidences of regulated expression levels on different substrates fermentation in strain CT7, which may lay the foundation for further genetic modification of other solventogenic strains to achieve efficient glycerol utilization.

## Results

### Glycerol-fed *Clostridium* sp. strain CT7 showed better butanol generation and bacterial growth capabilities than glucose

**Fig. 1:**
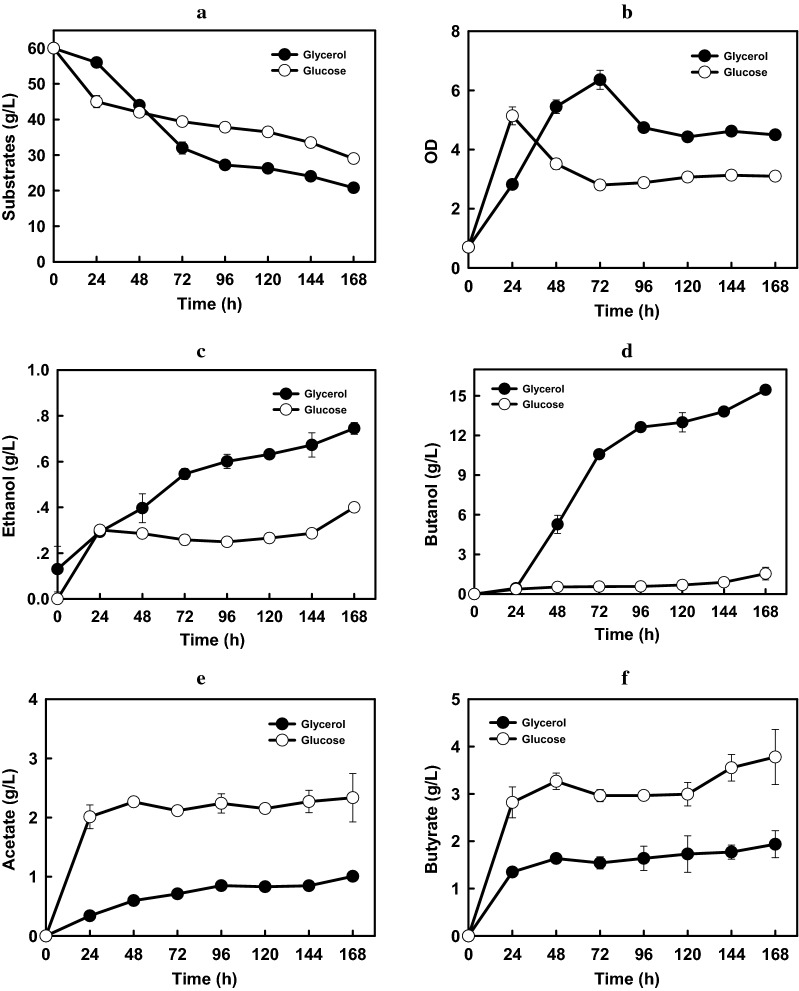
The comparison of butanol production between glycerol and glucose in *Clostridium* sp. strain CT7. Fermentation profiles comparisons in the consumption of carbon sources (**a**), the OD_600_ of the strain CT7 (**b**), the ethanol (**c**), butanol (**d**), acetate (**e**) and butyrate (**f**) production in strain CT7 fed with glycerol and glucose

When 60 g/L of glycerol was fed as the sole carbon source, the final production of butanol and VFAs obtained by *Clostridium* sp. strain CT7 were measured through the gas chromatography analysis. 16.60 g/L of butanol was generated by strain CT7 accompanied with 0.75, 1.01, 1.94 g/L of ethanol, acetate and butyrate, respectively, which represents the relatively high butanol production from glycerol by using wild type solventogenic *Clostridia* (Fig. 1). Impressively, different from the typical ABE fermentation profile from monosaccharides, much higher butanol ratio (95.68%) was achieved, which was attributed to the elimination of by-products acetone and 1,3-PDO. It is well known that ABE fermentation process could be divided into two phases, in which the first is acids formation phase and followed by solvents formation with reutilization of acids at the second phase. Whereas, the division of acidogenesis to solventogenesis was not obviously shown in strain CT7, as reflected by the constant VFAs levels when solventogenesis was still not initiated. After 24 h, the glycerol consumption rate reached 0.50 g/(L·h) corresponding to a sharp increase of butanol production from 0.44 g/L to 10.58 g/L with 0.21 g/(L·h) of butanol productivities. After 72 h, glycerol consumption and butanol production rates were both decreased, and butanol production was increased steadily.

**Fig. 2:**
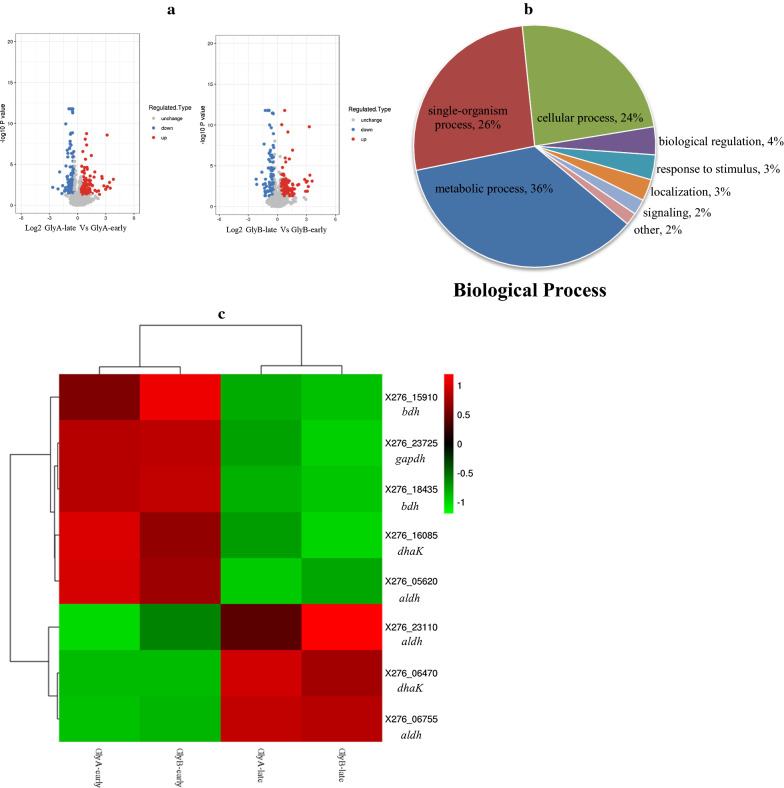
The comparison of proteins changes through the proteomics experiment at different fermentation time in *Clostridium* sp. strain CT7 fed with glycerol. **a** Volcano plots of proteins comparison between the different stages in strain CT7 fed with glycerol (the biological results were repeated twice). **b** Proteins related with biological process detected through the proteomics experiment which were classified using the Gene Ontology (GO) classification method. **c** Regulation profile of the selected important proteins related with butanol production from glycerol at different stages in strain CT7. The hierarchical clustering was used column and row clustering, and the legend represents the log2 fold-change values of genes’ expression

To better elaborate the underlying mechanism for efficient butanol production from glycerol, proteomic analysis was investigated, and culture samples were collected at 48 h and 72 h, representing the early and late fermentation stages, respectively. In total, 1840 proteins were identified, of which 1516 proteins were quantitated. Compared with those at 48 h, 142 proteins were regulated at 72 h based on the specified threshold criteria (1.3-fold), including 72 up-regulated and 70 down-regulated proteins (Fig. 2a). The Gene Ontology (GO) is a major bioinformatics initiative to unify the representation of genes. Based on the GO terms level 1 of biological process, approximately 36% of regulated proteins were involved in the metabolic process, suggesting that higher metabolic differences occurred in different fermentation phases (Fig. 2b). As seen in Fig. 2c, several proteomic expression levels occurring in butanol synthetic pathway were significantly changed. Among these, dihydroxyacetone kinase (DHAK) and glycerol dehydrogenase (GLD) responsible for glycerol utilization were highly up-regulated, whose proteomic expression levels were increased by 9.29 and 6.57-folds, respectively. While proteomic expression levels of glyceraldehyde-3-phosphate dehydrogenase (GAPD) and two butanol degydrogenases were down regulated by 0.69, 0.73 and 0.69-folds, respectively. The down-regulation of these genes may result in the decrease of butanol-production at 72 h than 48 h, which was consistent with fermentation results (Figs. 1 and 2c).

**Fig. 3:**
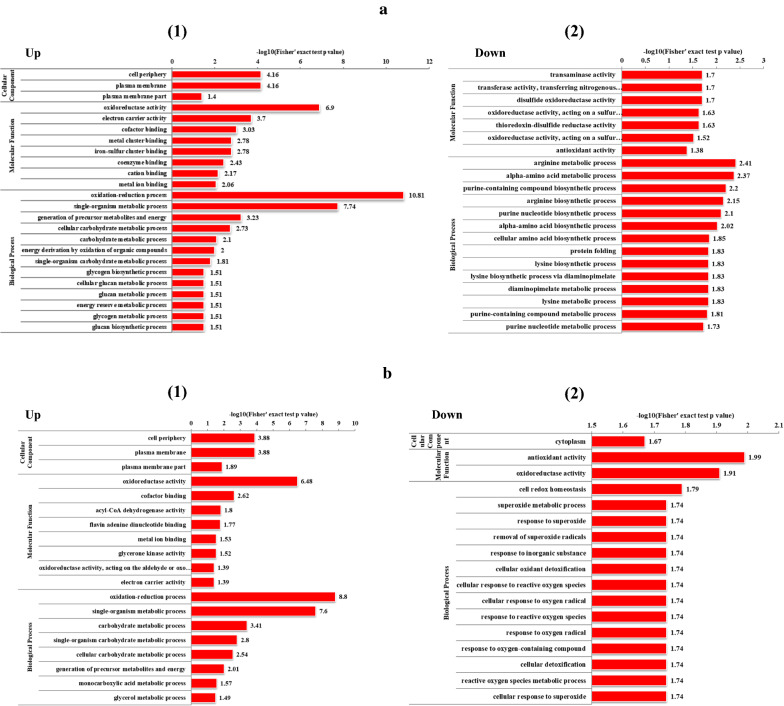
The comparison of proteins changes through the proteomics experiment at different carbon sources in *Clostridium* sp. strain CT7 using GO enrichment classification method. **a**, **b** The early and late fermentation stages, respectively. (1) and (2) represent the up-regulation and down-regulation in the different stages, respectively

**Fig. 4:**
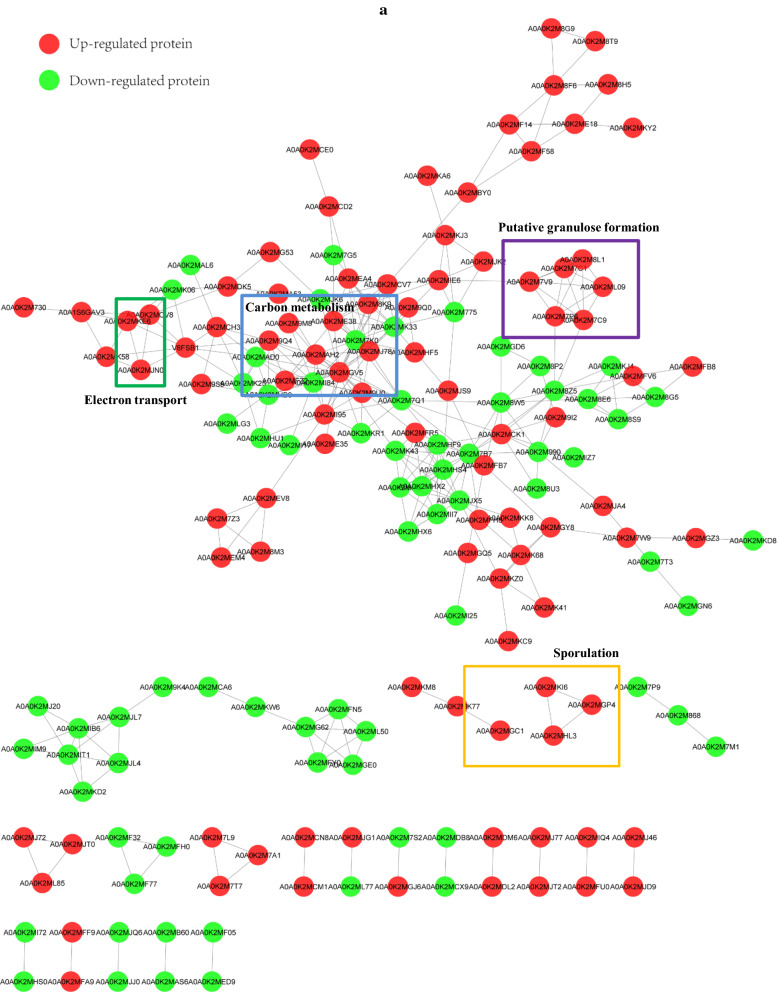

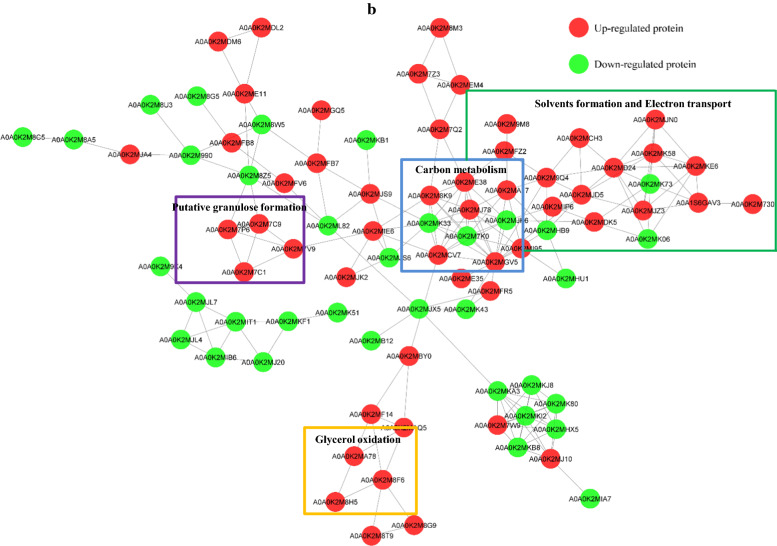
The protein-protein interaction network of changed proteins fed with glycerol and glucose in *Clostridium* sp. strain CT7 at the early (**a**) and late (**b**) fermentation stages. The red circles represented the up-regulated proteins and the green circles were those down-regulated ones

Another interesting phenomenon was that strain CT7 showed preference of glycerol over glucose. When 60 g/L of glucose was utilized as the carbon source, only 1.80 g/L of butanol was produced accompanied with 2.34 g/L of acetate and 3.78 g/L of butyrate. Thus, compared with using glycerol as the carbon source, much lower butanol (1.80 g/L Vs 16.60 g/L) and higher VFAs (6.12 g/L Vs 2.95 g/L) production were observed from glucose (Fig. 1). As known, glucose catabolism can generate ATP energy through VFAs production process, mainly acetate and butyrate in this case. The nearly doubled VFAs production will provide higher energy source for bacterial growth of strain CT7. Indeed, glucose consumption reached the fastest level within the first 24 h when glucose was used as carbon source. Cell density (OD_600_) could also reach the highest 5.14. Higher OD_600_ was observed from glucose amended medium accompanied with higher VFAs production, further implying that glucose-fed medium could more favor VFAs production and cell growth. After 24  h, glucose consumption was decreased sharply with the sluggish increase of products, accompanying with even a decrease of cell density of strain CT7. It should also be noticed that a relatively longer lag phase under glycerol media was observed as reflected by a lower glycerol consumption and later growth cessation within first 24 h. According to the iTRAQ data, 364 and 228 regulated proteins were identified (P  < 0.05, fold change 1.3) in the early and late fermentation stages from 60 g/L of glycerol, respectively. Among these proteins, 192 and 172 proteins were up- and down-regulated in the early fermentation stage, while 137 and 91 proteins were up- and down-regulated in the late fermentation stage. As shown in Fig. 3, most protein classes related with butanol production, such as carbohydrate metabolic process and electron carrier activity etc. were up-regulated in glycerol-fed medium. The protein interaction network shown in Fig. 4 also suggested the strong relationship of different functional proteins, and the obvious up-regulation of functional proteins mainly focused on the carbon metabolism, solvent production, electron-transferring and stress response etc.

### Identification of up-regulated proteins playing major roles in glycerol utilization within strain CT7

**Fig. 5:**
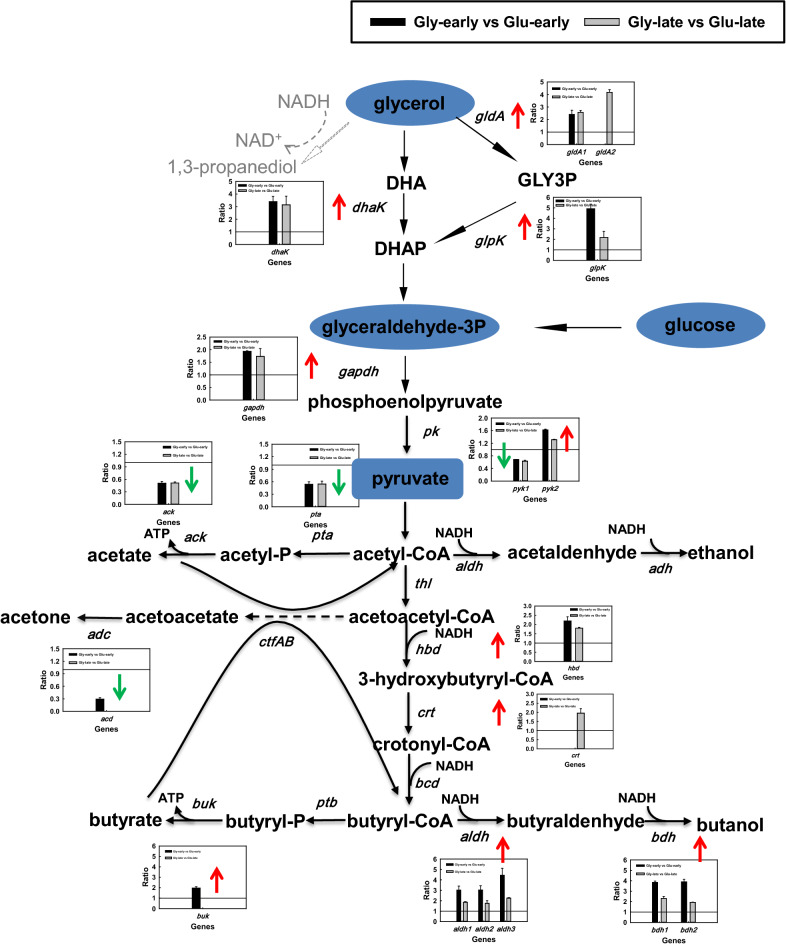
Metabolic pathway of glycerol and glucose bioconversion to butanol in *Clostridium* sp. strain CT7

In order to better elaborate the response of strain CT7 towards glycerol and glucose, the proteomic expression levels related with butanol generation process under different fermentation stages measured by iTRAQ strategy were compared. Through the analysis of proteomic expression results of strain CT7, two glycerol dehydrogenases and one dihydroxyacetone kinase (DHAK) may play essential roles in the glycerol metabolism, which were up-regulated under glycerol-fed medium. GLD encoded by A0A0K2ME18 showed 2.40-folds up-regulation in the early fermentation stage, and 2.58-folds up-regulation in the late fermentation stage. However, the other GLD encoded by A0A0K2MA78 showed 4.18-folds up-regulation only in the late fermentation stage. In addition, the proteomic expression level of DHAK encoded by A0A0K2MF58 was found to have 3.40- and 3.15-folds increase at early and late fermentation stages, respectively. Taken together, proteomic expression levels related with glycerol metabolism were all improved throughout the fermentation phages (Fig. 5).

Apart from the unique glycerol utilization proteins, A0A0K2ME38 encoding glyceraldehyde-3-phosphate dehydrogenase showed 1.93- and 1.73-folds up-regulation under glycerol media at early and late fermentation stages, respectively. Furthermore, two pyruvate kinases (PYK) were identified, which showed different proteomic expression profiles. A0A0K2MJ78 had an up-regulation of 1.62-folds and 1.31-folds at early and late fermentation stages, respectively. However, A0A0K2M7K0 encoding PYK with molecular size of 50.5 kDa exhibited the down-regulation in these two different fermentation phases. Another interesting phenomenon was that a protein responsible for glucose utilization encoding ATP-dependent 6-phosphofructokinase (A0A0K2MIE6), also exhibited up-regulation in the glycerol-fed medium. However, proteins capable of metabolizing other carbohydrates, like xylose isomerase were dramatically down-regulated in strain CT7.

### Proteomic analysis of pyruvate acetyl-CoA node, a focal point in the metabolism for clostridia

In anaerobes, pyruvate is oxidized to CO_2_ and acetyl-CoA. Pyruvate: ferredoxin/flavodoxin oxidoreductase (PFOR) is mainly responsible for the oxidative decarboxylation of pyruvate in many anaerobic bacteria, which contains thiamin pyrophosphate (TPP) for cleaving carbon-carbon bonds next to the carbonyl group, as well as Fe-S clusters for electron transfer [17, 19, 20]. Through the proteomic analysis, three PFORs were identified within strain CT7, which possessed 56, 4 and 9 unique peptides compared to homologue proteins X276_03990, X276_17585 and X276_19390 encoding PFORs from *C. beijerinckii* NRRL B-598. However, only A0A0K2MGV5 with molecular size of 129.6 kDa was regulated, showing 2.40- and 1.67-folds up-regulation in the early and late fermentation stages, respectively.

In most clostridia, Fd_ox_ regeneration via hydrogenase accompanies with reducing power generation, which is strongly associated with butanol production. Hydrogenase is one of key enzymes for hydrogen production in clostridia by transferring electrons from ferredoxins and flavodoxins to protons, which can be divided into [NiFe] and [FeFe] hydrogenases based on their metaollocenter composition [21]. Through the proteomic analysis, A0A0K2M8M5, one of the identified hydrogenases belonging to [FeFe] hydrogenase showed 1.82-folds higher proteomic expression level at 48 h under glycerol medium. Accompanied with the decreased gas production after the stationary phase, the [FeFe] hydrogenase exhibited down-regulated proteomic expression level (0.68-folds) in the late fermentation stage of glycerol-fed strain CT7, resulting in the similar proteomic expression levels between glycerol- and glucose-fed ones. In addition, six electron-transferring flavoproteins (ETF) were also identified, which served as electron carriers [22]. A0A0K2MK06 and A0A0K2MK73 showed higher proteomic expression levels under glucose medium at both early and late fermentation phases. On the contrary, A0A0K2MJN0 and A0A0K2MKE6 showed higher proteomic expression levels under glycerol ones. Another two electron-transferring flavoproteins A0A0K2MD99 and A0A0K2MCV8 only showed up-regulated proteomic expression levels in the early fermentation stage under glycerol media than glucose ones.

### Regulation analysis for conversion of acetyl-CoA/butyryl-CoA to solvents and VFAs

Four enzymes, including thiolase (THL), β-hydroxybutyryl-CoA dehydrogenase (HBD), crotonase (CRT) and butyryl-CoA dehydrogenase (BCD) are involved in the catalysis from acetyl-CoA to butyryl-CoA. Among these enzymes in strain CT7, only the expression level of HBD was up-regulated with 2.20-folds increase in the early fermentation stage under glycerol-fed media. HBD and CRT both showed higher proteomic expression levels in the late fermentation stage. In addition, as shown in Figs. 0 and 10.21 g/(L·h) of butanol production productivity from glycerol was present at 48 h, which was obviously higher than that from glucose. Consistent with fermentation results, the identified three aldehyde dehydrogenases (ALDH) and two butanol dehydrogenases (BDH) in strain CT7 all exhibited up-regulation under glycerol media. ALDHs showed 3.03-, 3.02- and 4.44-folds increased proteomic expression levels, and BDHs showed 3.85- and 3.90-folds increased proteomic expression levels, respectively. In the late fermentation stage, these five proteins showed higher proteomic expression levels under glycerol medium ranging from 1.8 to 2.5 folds, however, the increased folds were still lower than in the early fermentation stage. It should also be noticed that although strain CT7 could not produce acetone due to the deficiency of *ctfAB*, the expression level of acetoacetate decarboxylase (ADC) was much lower from glycerol, which was one of major enzymes related to the acetone generation.

Doubled VFAs production was observed under glucose media than glycerol. Through the proteomics analysis, down-regulated proteomic expression levels of phosphate acetyltransferase (PTA) and acetate kinase (ACK) occurred in the whole fermentation phases, which showed 0.5-folds down-regulation. The proteomic expression level of phosphate butyryltransferase (PTB) showed the similar downtrend (0.61-folds). A putative butyrate kinase showed a crosscurrent with 1.97-folds increase under glycerol media. While other two butyrate kinases maintained the similar proteomic expression levels from different substrates.

## Glycerol utilization may strengthen the trigger of sporulation response

To survive in some adverse conditions, sporulation is a commonly adopted strategy for some phylum firmicutes, like *Clostridium* and *Bacillus*. Physical and chemical stresses, such as the existence of solvents, oxidizing agents, lytic enzymes may trigger the initialization of sporulation process, which has been proved as a threshold for the switch from the acidogenic to solventogenic metabolism [6]. The master regulator of sporulation, Spo0A was speculated to affect solvent production through controlling genes expression, such as alcohol/aldehyde dehydrogenases [23, 24]. Proteomic expression levels of eight proteins associated with sporulation process including Spo0A were highly increased in the early fermentation stage under glycerol medium. Specifically, proteomic expression level of A0A0K2MKI6 encoding stage V sporulation protein was up-regulated by 7.90-folds in the early fermentation stage. In the late fermentation stage, proteomic expression levels of sporulation related proteins were all decreased. However, five of these proteins were still obviously up-regulated in the glycerol medium than in glucose one. Furthermore, the proteomic expression level of a histidine kinase was increased by 1.67-folds at 48 h, which might response to the environmental signal for directly activating Spo0A [25, 26]. These results suggested a plausible tendency of the glycerol-fed cultures to initiate a slightly earlier sporulation response than glucose-fed ones, which may be associated with butanol production.

## Discussion

Different from the most well-known solventogenic *C. acetobutylicum* and *C. beijerinckii*, *Clostridium* sp. strain CT7 showed the preference for glycerol over monosaccharides as the substrate. 16.60 g/L of butanol was generated, representing the highest butanol production from glycerol in the batch mode. Attributing to the deficiency of key genes responsible for acetone and 1,3-PDO production, uncoupled by-products fermentation mode significantly improved the butanol ratio in strain CT7. Proteomics analysis further proved: (i) efficient capability of glycerol utilization may be attributed to the high expression level of proteins in glycerol oxidation and glycolysis pathway, such as glycerol dehydrogenases, dihydroxyacetone kinase, glyceraldehyde-3-phosphate dehydrogenase, pyruvate kinases etc.; (ii) lower proteomic expression levels of phosphate acetyltransferase, acetate kinase, phosphate butyryltransferase etc. would lead to less VFAs production; (iii) up-regulation of butyryl-CoA and solvents formation proteins, including β-hydroxybutyryl-CoA dehydrogenase, crotonase, aldehyde dehydrogenases and butanol dehydrogenases resulted in higher butanol production.

Strong electron transfer system would benefit for higher butanol production under glycerol-fed strain CT7. Pyruvate ferredoxin/flavodoxin oxidoreductase can transfer the electrons generated in the decarboxylation reaction from pyruvate to reduced ferredoxin (Fd_red_) [27]. Different substrates may employ different electron acceptors for PFORs. As the proteomic expression levels of flavodoxin were both increased in the early (1.50-folds) and late fermentation (1.95-folds) stages, strain CT7 probably employed flavodoxin as electron acceptor PFOR instead of ferredoxin under glycerol media. The obvious down-regulation of ferredoxin at 48 h indicated that ferredoxin was the preferred electron acceptor for strain CT7 in glucose-fed ones. Additionally, Fd_ox_ regeneration is the main metabolic pathway for H_2_ production via hydrogenase accompanied with reducing power regeneration in most clostridia, which may further help boost butanol production [17]. The higher expression level of [FeFe] hydrogenase in the early growth phase was consistent with the higher H_2_ production, indicating that more reducing power may be generated under glycerol medium. In addition, the electron transfer flavoprotein serves as an obligatory electron carrier from various dehydrogenases to the electron transport chain. ETF is a heterodimer consisting of an alpha and a beta subunit, which will bind one molecule of FAD per dimmer [28]. The up-regulated electron transfer flavoproteins would play key roles in the redox process and feed electrons to ferredoxin. Moreover, many electron transport complex subunits exhibited the increased tendency from glycerol than glucose, especially in the early fermentation phase. In strain CT7, the proteomic expression level of A0A0K2ME53 encoding electron transport complex subunit D showed striking 5.47-folds boost in the early fermentation phase, indicating the stronger electron transport under glycerol media. These proteins are constituently expressed to stabilize the internal redox balance and provide reducing power for butanol production.

**Fig. 6:**
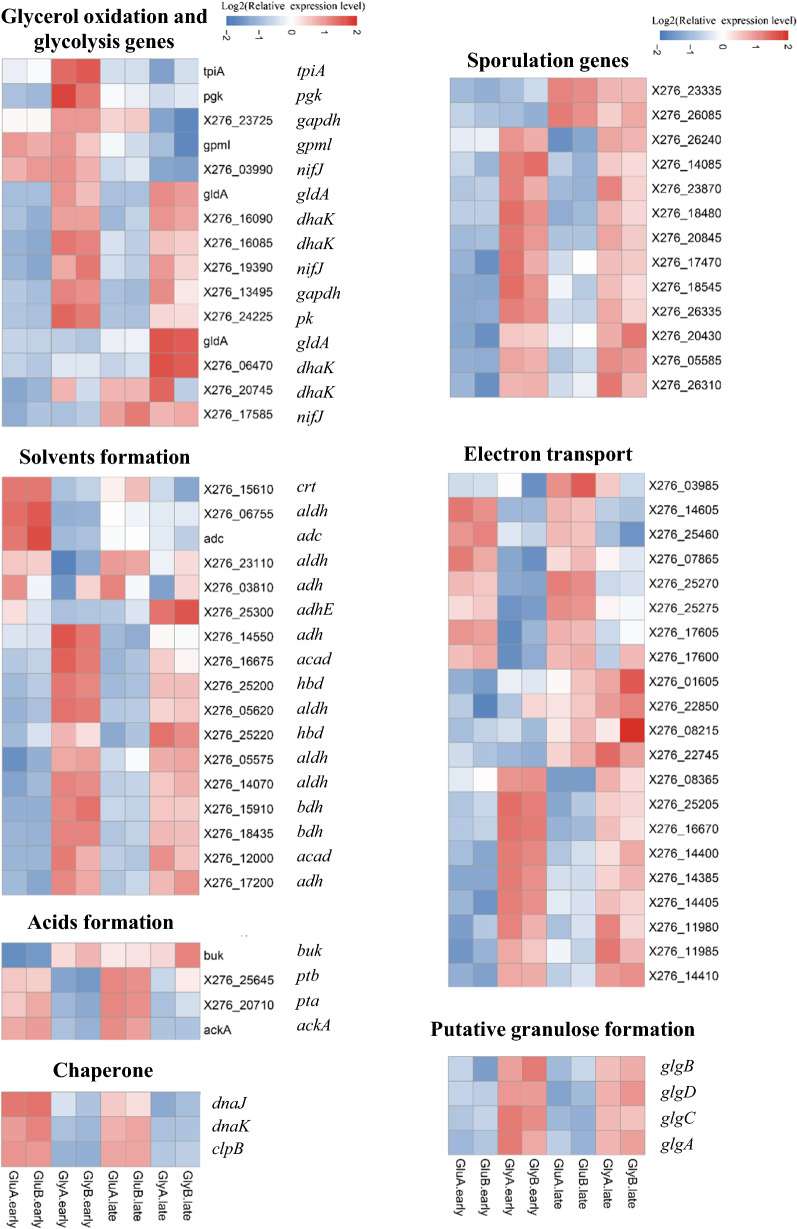
Comparison expression profiles of key proteins related with butanol production fed with glycerol and glucose in different time course

To survive in the adverse environment, several stress responses would be triggered in *Clostridia*. Granulose is a glycogen-like polymer to reserve carbon and energy sources under severe environment conditions [29]. Several putative granulose formation proteins were up-regulated in glycerol-fed strain CT7, including glycogen synthase (GLGA), 1,4-alpha-glucan branching enzyme (GLGB), glucose-1-phosphate adenylyltransferase (GLGC) and ADP-glucose pyrophosphorylase (GLGD) (Fig. 6). In addition, Spo0A is a common transcription factor, which controls the transition of bacterium into spore form. As described in previous studies, overexpression of Spo0A in *C. acetobutylicum* increased bacterial tolerance and prolonged metabolism in response to butanol stress [24, 30]. In addition, higher solvents and lower VFAs production were obtained in Spo0A overexpression one. The higher expression levels in solvent formation genes indicated that Spo0A is necessary for the normal solventogenic process by controlling these solvent formation genes. The stronger induction of sporulation in strain CT7 further confirmed the correlation between sporulation and solvent production processes.

In addition, the proteomic expression levels of other stress response proteins also changed under glycerol media. Chaperones were found to be expressed in response to stresses within many bacteria, which play essential roles in the folding and/or assembly of proteins [31, 32]. Interestingly, chaperone proteins DnaK and ClpB were both down-regulated in glycerol-fed strain CT7. Especially, the expression levels of ClpB were decreased by 0.36 and 0.46-folds in the early and late fermentation phases, respectively. In addition, the phage shock protein (psp) system is a specialized extracytoplasmic stress response one through lowering proton motive force (PMF) within bacteria. Psp system has been reported to be the pivotal component of signal transduction pathway, which helps to maintain the integrity of cytoplasmic membrane and interact with proteins responsible for proton transportation using proton gradient or ATP [33–35]. The down-regulation of pspA in glycerol-fed strain CT7 was observed at both early and late fermentation stages, indicating that more psp proteins were induced in glucose-fed ones to resist the environmental stress, maintain the integrity of cytoplasmic membrane and restore the desired PMF. Accompanied with up-regulated Spo0A, proteins related with stress response also showed higher expression levels. The crosscurrent phenomenon observed in strain CT7 indicated that Spo0A may possess different inducing mechanism from other stress response proteins.

## Conclusions

To better understand the mechanism for higher butanol production from glycerol over glucose by newly isolated solventogenic *Clostridium* sp. strain CT7, the proteomics analysis through iTRAQ technology was performed. The up-regulation of proteins expression levels in glycerol oxidation and glycolysis pathway, including glycerol dehydrogenases, dihydroxyacetone kinase, glyceraldehyde-3-phosphate dehydrogenase, pyruvate kinases etc., and solvents formation proteins, especially aldehyde dehydrogenases and butanol dehydrogenases may attribute to the enhancement of butanol production from glycerol than glucose by strain CT7. The higher reducing power was obtained owing to the stronger electron transport and acceptor capacity in glycerol-fed strain CT7, which would also help to boost the butanol production. In addition, the sporulation response was possibly beneficial for solvent production, and the higher proteomic expression levels of histidine kinase and sporulation proteins supported the hypothesis that stronger sporulation response was present in glycerol-fed strain CT7. These analysis data would benefit for designing efficient solvent producing microorganisms from glycerol medium.

## Methods

### Bacteria strain, culture medium and growth conditions

*Clostridium* sp. strain CT7 was cultivated anaerobically at 37 °C with a shaking speed of 150 rpm in mineral salts medium amended with glycerol or glucose as the carbon source. The ingredients of defined mineral salts medium were (g/L): KH_2_PO_4_, 0.75; K_2_HPO_4_, 0.75 and yeast extract, 5. In addition, 1 mL of salts medium, 1 mL of trace element solution, 1 mL of Na_2_SeO_3_-Na_2_WO_4_ solution and 10 mg of resazurin were added to 1 L of the medium. Then the medium was purged with N_2_ and added reductants. Subsequently, 20 mM 2-(N-Morpholino) ethanesulfonic acid (MES) was added to adjust its initial pH to 6.0. The detailed culture medium and growth conditions were previously described by Xin et al. (2017). After the inoculation of strain CT7, the bacterial cultures were maintained at pH 5.5 by addition of 3 M NaOH solution.

For the proteomic analysis, 25 mL cultures were collected at two different time-points: first at 48 h, which is the early fermentation phase with exuberant bacterial growth and products productivity; and second at 72 h, which is the late fermentation phase of the culture with basial growth cessation and lower productivity. To remove the extracellular proteases, cells were washed by PBS buffer for three times and stored at -80 °C freezer.

### Analytic method

Cell concentration, glycerol and glucose consumption, solvents and acids generation were analyzed for the fermentation profiles. Cell concentration was measured at 600 nm of optical density with appropriate dilution. Concentrations of glycerol and glucose were determined by high-performance liquid chromatography (HPLC) (UitiMate 3000 HPLC system, Dionex, USA) using an ion exchange chromatographic column (Bio Rad Aminex HPX-87H column, USA) at a wavelength of 215 nm on a UVD 170U ultraviolet detector. The operating conditions were as following: temperature, 55 °C; mobile phase, 5 mM H_2_SO_4_; flow rate, 0.6 mL/min. Solvents and acids were measured by gas chromatography (GC) analysis as previously reported [18]. Iso-butanol was used as the internal standard.

### Protein extraction and iTRAQ labeling

The following process of quantitative proteomic through iTRAQ was performed by Jingjie PTM BioLab (Hangzhou) Co. Ltd. The collected cells were re-suspended in a lysis buffer (8 M urea, 1% Protease Inhibitor Cocktail). Obtained debris were separated by centrifugation (12,000*g*) at 4 °C for 10 min. The supernatant protein concentration was measured with BCA kit according to manufacturer’s protocol. Then, the protein solution was reduced with 5 mM dithiothreitol for 30 min at 56 °C and alkylated with 11 mM iodoacetamide for 15 min in darkness. Finally, trypsin was added to digest proteins.

Obtained peptides through trypsin digestion were desalted by Strata X C18 SPE column (Phenomenex) and vacuum-dried, and then reconstituted in 0.5 M TEAB by TMT kit/iTRAQ kit according to manufacturer’s protocol. The biological replicates of early glucose-fed sample 1 and sample 2 were labeled with reagents 119 and 121; and the late glucose-fed of sample 1 and sample 2 with were labeled with reagents 115 and 116. In the glycerol-fed ones, the early fermentation samples were labeled with reagents 117 and 118, and the late fermentation samples were labeled with reagents 113 and 114, respectively.

### HPLC fractionation and LC–MS/MS analysis

The tryptic peptides were fractionated into fractions by high pH reverse-phase HPLC using Agilent 300 Extend C18 column. These peptides were first separated with a gradient of 8–32% acetonitrile (pH 9.0) over 60 min into 60 fractions. Then, the peptides were combined into 18 fractions. The dried peptides by vacuum centrifuging were dissolved in formic acid and loaded onto a home-made reversed-phase analytical column. The mobile phases were solvents composed of 0.1% formic acid in 98% acetonitrile at a flow rate of 400 nL/min on as EASY-nLC 1000 UPLC system. Peptides were separated from the increased gradient, and the process was ranged from 6 to 23% over 26 min, 23–35% in 8 min, then climbing to 80% in 3 min and holding at 80% for 3 min.

The peptides were subjected to NSI source followed by tandem mass spectrometry (MS/MS) in Q Exactive™ Plus (Thermo) coupled online to the UPLC. The applied capillary voltage was 2.4 kV. The m/z scan range was 350 to 1550 for full scan, and intact peptides were detected in the Orbitrap at a resolution of 60,000. Peptides were then selected for MS/MS, fixed first mass was set as 100 m/z and the fragments were detected in the Orbitrap at a resolution of 15,000. A data-dependent procedure that alternated between one MS scan followed by 20 MS/MS scans with 30.0 s dynamic exclusion. Automatic gain control (AGC) was set at 5E4 and an absolute threshold of 5,000. Finally, the resulting MS/MS data were processed using Maxquant search engine (v.1.5.2.8). Trypsin/P was specified as cleavage enzyme allowing up to 2 missing cleavages. The mass tolerance for precursor ions was set as 20 ppm in First search and 5 ppm in Main search, and the mass tolerance for fragment ions was set as 0.02 Da. Tandem mass spectra were searched against *C. beijerinckii* NRRL database, and annotated at the common databases, including GO annotation, Kyoto Encyclopedia of Genes and Genomes (KEGG) database etc. The quantitative value of the peptide in each sample is calculated based on the ratio of the labeling reporter ion intensities in MS/MS spectra from raw data sets. Additionally, protein function enrichment analysis was performed in R by fisher.test function of stats package. The mass spectrometry proteomics data have been deposited to the ProteomeXchange Consortium via the PRIDE partner repository with the dataset identifier PXD016848.

### The construction of protein–protein interaction network

All differentially expressed protein name identifiers were searched against the STRING database version 11.0 for protein-protein interactions. To exclude the external candidates, only interaction proteins belonging to the searched data were selected. “confidence score” in STRING was defined as the interaction confidence, and a confidence score ≥ 0.7 (high confidence) was fetched. Interaction network form STRING was visualized by using Cytoscape. The molecular complex detection (MCODE), a part of the plug-in toolkit of the network analysis and visualization software Cytoscape was used to analyze the dense connected regions.

## Data Availability

Not applicable.
